# Supplementary material for: Causal Relationship between Aging and Anorexia Nervosa: A White-Matter-Microstructure-Mediated Mendelian Randomization Analysis
Source: Biomedicines. 2024 Aug 16;12(8):1874. doi: 10.3390/biomedicines12081874 (PMC11351342; doi:10.3390/biomedicines12081874)
Supplement: Supplementary file 1 [file biomedicines-12-01874-s001.zip › Supplementary Figure/Figure S4/Singe SNP plot of MR analysis for TL to ubm-b-1743.pdf]

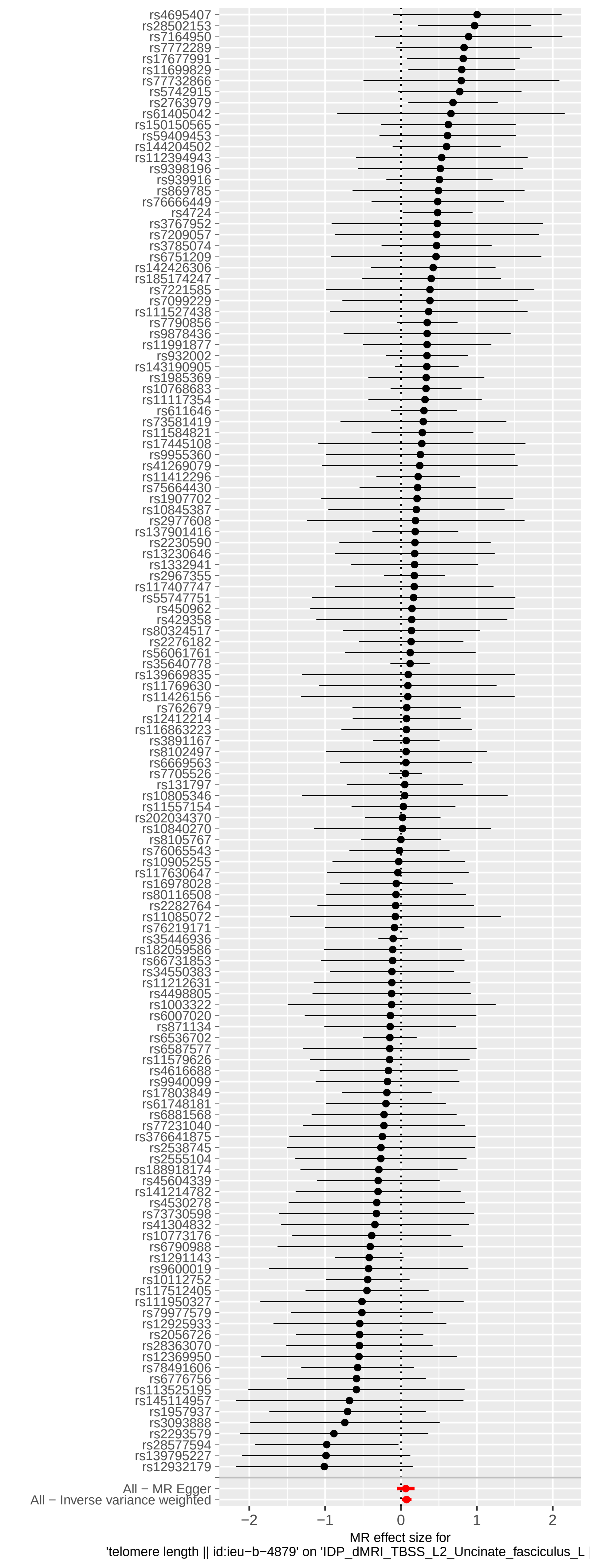
rs4695407
rs28502153
rs7164950
rs7772289
rs17677991
rs11699829
rs77732866
rs5742915
rs2763979
rs61405042
rs150150565
rs59409453
rs144204502
rs112394943
rs9398196
rs939916
rs869785
rs76666449
rs4724
rs3767952
rs7209057
rs3785074
rs6751209
rs142426306
rs185174247
rs7221585
rs7099229
rs111527438
rs7790856
rs9878436
rs11991877
rs932002
rs143190905
rs1985369
rs10768683
rs11117354
rs611646
rs73581419
rs11584821
rs17445108
rs9955360
rs41269079
rs11412296
rs75664430
rs1907702
rs10845387
rs2977608
rs137901416
rs2230590
rs13230646
rs1332941
rs2967355
rs117407747
rs55747751
rs450962
rs429358
rs80324517
rs2276182
rs56061761
rs35640778
rs139669835
rs11769630
rs11426156
rs762679
rs12412214
rs116863223
rs3891167
rs8102497
rs6669563
rs7705526
rs131797
rs10805346
rs11557154
rs202034370
rs10840270
rs8105767
rs76065543
rs10905255
rs117630647
rs16978028
rs80116508
rs2282764
rs11085072
rs76219171
rs35446936
rs182059586
rs66731853
rs34550383
rs11212631
rs4498805
rs1003322
rs6007020
rs871134
rs6536702
rs6587577
rs11579626
rs4616688
rs9940099
rs17803849
rs61748181
rs6881568
rs77231040
rs376641875
rs2538745
rs2555104
rs188918174
rs45604339
rs141214782
rs4530278
rs73730598
rs41304832
rs10773176
rs6790988
rs1291143
rs9600019
rs10112752
rs117512405
rs111950327
rs79977579
rs12925933
rs2056726
rs28363070
rs12369950
rs78491606
rs6776756
rs113525195
rs145114957
rs1957937
rs3093888
rs2293579
rs28577594
rs139795227
rs12932179
All − MR Egger
All − Inverse variance weighted
−2
−1
0
1
2
MR effect size for
'telomere length || id:ieu-b-4879' on 'IDP_dMRI_TBSS_L2_Uncinate_fasciculus_L |
